# Moral Intelligence and Ethical Decision‐Making Among Nurses: Moderating Mediation Roles of Ethical Responsibility and Time Urgency

**DOI:** 10.1155/jonm/3767394

**Published:** 2026-10-26

**Authors:** Ethel Yiranbon Annor-Tenkorang, Prince Addai, Rejoice Mottey, James Abadektemi Abasimi, Emmanuel Atta Kumah Amponsah

**Affiliations:** ^1^ Ghana Communication Technology University Accra Ghana site.gctu.edu.gh

**Keywords:** ethical decision-making, ethical responsibility, Ghana, moral intelligence, nurses, time urgency

## Abstract

**Purpose:**

Ethical decision‐making remains a critical concern within the nursing profession due to the moral complexities and time‐sensitive nature of healthcare delivery. Although moral intelligence has been recognised as an important ethical resource in organisational settings, the intervening mechanisms that influence the relationship among nurses remain inadequately explored. This study assesses the mediating role of ethical responsibility in the relationship between moral intelligence and ethical decision‐making among nurses. The study further investigates the moderating role of time urgency in the mediated relationship.

**Design:**

The study collected data from 261 nurses using a multistage sampling technique. Questionnaires were completed anonymously through a time‐lagged survey method. Process Macro was utilised to test the proposed moderated mediation model.

**Results:**

Results indicate that moral intelligence positively relates to ethical decision‐making through ethical responsibility. Furthermore, time urgency moderates the indirect association between moral intelligence and ethical decision‐making through ethical responsibility such that high time urgency weakened the mediated relationship.

**Contribution:**

This study advances ethical decision‐making research by explaining how moral intelligence relates to nurses’ ethical decision‐making through ethical responsibility and under varying levels of time urgency. It also extends evidence on ethical decision‐making within the African healthcare context.

**Implication for Nursing Management:**

Nursing managers should strengthen nurses’ moral intelligence and ethical responsibility through ethics‐focused training and supportive leadership. Given that time urgency weakens the indirect effect of moral intelligence on ethical decision‐making, managers should also minimise unnecessary time pressure and provide nurses with sufficient opportunities for ethical reflection when making complex clinical decisions.

## 1. Introduction

Nursing professionals routinely encounter situations that demand difficult moral judgements while operating in highly dynamic healthcare environments [1]. Decisions related to patient care often involve competing priorities, including patient safety, institutional regulations, professional ethics and limited medical resources. Because nurses are directly involved in continuous patient interaction, they are frequently required to respond quickly to ethically sensitive situations, such as treatment fairness, confidentiality concerns, informed consent and end‐of‐life care [2]. In Ghana, these ethical demands occur within a healthcare system characterised by increasing patient needs, resource constraints and pressure on healthcare professionals to deliver quality care despite operational challenges. Public healthcare facilities, where nurses constitute a critical component of service delivery, often require practitioners to balance professional responsibilities with workload demands and limited resources [3]. These realities make ethical decision‐making a fundamental component of effective nursing practice and highlight the importance of identifying factors that strengthen ethical judgement among nurses [1].

One factor that has attracted growing academic interest in ethical conduct is moral intelligence. Nurses are expected to maintain ethical behaviour despite emotional exhaustion, operational stress and demanding clinical responsibilities. Individuals with stronger moral intelligence are capable of aligning their actions with ethical principles even under pressure [4]. Although earlier studies have associated moral intelligence with desirable organisational outcomes [5, 6], insufficient attention has been given to its association with ethical decision‐making specifically within nursing settings. Moreover, although previous studies have suggested an association between moral intelligence and ethical behaviour, the internal processes through which moral intelligence relates to ethical decision‐making remain insufficiently understood, particularly among healthcare professionals in Sub‐Saharan Africa. Addressing this gap, the present study proposes ethical responsibility as an important internal process linking moral intelligence to ethical decision‐making. Nurses who possess elevated levels of moral intelligence experience a stronger sense of ethical obligation, which may encourage more ethically sound decisions during challenging clinical situations [7].

Moreover, ethical decisions in healthcare are rarely made under ideal conditions. Nurses commonly work in environments characterised by heavy workloads, urgent patient needs and strict time constraints [1]. Such conditions may interfere with thoughtful ethical evaluation and reduce the ability to carefully consider the consequences of decisions. Time urgency represents a major environmental pressure in healthcare settings. Nurses who frequently experience time urgency are susceptible to cognitive overload and psychological exhaustion, factors that can compromise effective ethical reasoning in clinical practice [8]. Consequently, the positive association between ethical responsibility and ethical decision‐making may diminish under conditions of severe time pressure, as individuals are required to make rapid judgements with limited opportunity for careful ethical consideration. Although workplace stressors have been discussed in prior ethical research [9], limited empirical evidence exists regarding the extent to which time urgency influences the association between moral intelligence and ethical decision‐making.

Based on the Social Cognitive Theory (SCT), the study argues that nurses with higher moral intelligence develop ethical responsibility, which subsequently enhances ethical decision‐making. However, the strength of this process may vary depending on contextual workplace conditions. Specifically, the study proposes that time urgency weakens the indirect relationship between moral intelligence and ethical decision‐making through ethical responsibility. Therefore, this study examines the relationship between moral intelligence and ethical decision‐making, the mediating role of ethical responsibility in this relationship and the moderating role of time urgency in the indirect relationship between moral intelligence and ethical decision‐making through ethical responsibility. Figure 1 summarises the study’s primary theoretical and practical contributions to the existing body of literature.

**FIGURE 1:**
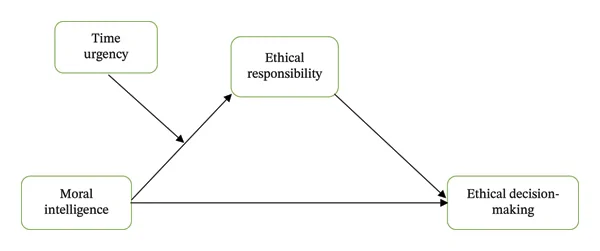
Conceptual relationships.

## 2. Review of Literature

### 2.1. Theoretical Underpinning

This study is anchored in SCT to explain how moral intelligence relates to ethical decision‐making among nurses through ethical responsibility under varying levels of time urgency. SCT posits that human behaviour is shaped through the reciprocal interaction of cognitive processes, behavioural patterns and environmental conditions [10]. The theory emphasises that individuals do not act solely based on external pressures or internal dispositions; rather, behaviour emerges from the continuous interaction between personal moral standards and situational influences [11]. From the perspective of SCT, moral intelligence represents a cognitive‐moral resource that guides behavioural regulation and ethical conduct. Individuals with high moral intelligence are more likely to evaluate situations based on moral principles such as integrity, responsibility, compassion and fairness [12]. These internal moral standards shape how nurses interpret ethical dilemmas and regulate their behaviour in clinical practice. The theory further suggests that moral cognition alone may not automatically produce ethical behaviour unless individuals internalise a sense of responsibility toward morally appropriate action [11]. In this regard, ethical responsibility reflects a self‐regulatory mechanism through which nurses translate moral understanding into ethical decision‐making. Nurses who possess strong ethical responsibility are more likely to feel accountable for the consequences of their actions and, therefore, demonstrate greater commitment to ethically appropriate decisions in patient care situations [13].

The theory further recognises that environmental and situational conditions influence whether cognitive moral capacities are effectively translated into behaviour [11]. Within healthcare environments, time urgency represents a significant contextual pressure that can shape ethical behaviour by limiting reflection, increasing psychological strain and constraining deliberate reasoning processes. Although nurses may possess high moral intelligence and strong ethical responsibility, excessive time pressure may reduce their ability to apply these moral standards in decision‐making [12]. Drawing from SCT, this study, therefore, argues that time urgency operates as a contextual condition that influences the extent to which moral intelligence and ethical responsibility translate into ethical decision‐making.

### 2.2. Moral Intelligence and Ethical Decision‐Making

Moral intelligence represents an individual’s internal capacity to understand, interpret and apply universal moral principles such as integrity, responsibility, compassion, and fairness in behavioural situations [14]. It reflects a cognitive‐moral competence that guides individuals in distinguishing right from wrong and aligning actions with ethical standards, particularly in complex and ambiguous situations [15]. In professional contexts such as nursing, moral intelligence becomes essential due to the frequency of ethically sensitive decisions involving patient welfare, confidentiality and life‐impacting judgements. According to Ellemers et al. [4], moral intelligence has been associated with a range of positive behavioural outcomes. However, one important outcome that has received comparatively limited empirical attention is ethical decision‐making. Ethical decision‐making refers to the cognitive and behavioural process through which individuals evaluate alternatives in morally charged situations and select actions that are consistent with accepted ethical principles and professional codes of conduct [16]. It reflects the importance of integrating both moral cognition and professional standards in guiding individuals’ responses to ethical challenges in the workplace [17].

According to SCT, behaviour is shaped through the dynamic interaction of personal cognitive factors, behavioural tendencies and environmental influences [10]. Moral intelligence functions as a key cognitive determinant that shapes how individuals interpret ethical situations and regulate their behaviour. Employees with high moral intelligence are more likely to possess strong internal moral standards that guide their evaluation of clinical dilemmas, enabling them to identify ethically appropriate courses of action [18]. Through self‐regulatory processes, such individuals are better able to align their decisions with moral expectations even in complex and high‐pressure healthcare environments. Moral intelligence enhances ethical decision‐making by strengthening moral awareness, improving ethical judgement, and reinforcing the motivation to act in accordance with ethical principles. Nurses with strong moral intelligence consider patient’s dignity, fairness and responsibility when making clinical decisions, thereby reducing the likelihood of unethical practices or moral lapses [18]. Existing literature has largely examined general ethical reasoning and professional values [5, 19], with limited attention given to moral intelligence as a distinct predictor of ethical decision‐making in nursing contexts. This gap highlights the need for empirical investigation into how moral intelligence relates to ethical decision‐making within healthcare practice. Accordingly, it is proposed that:

H_1_: Moral intelligence positively relates to ethical decision‐making.

### 2.3. Ethical Responsibility as a Mediator

Ethical responsibility represents a psychological and professional disposition characterised by an internalised obligation to act in accordance with moral principles [20]. It reflects an individual’s commitment to fulfilling moral duties and adhering to professional ethical standards [7]. Ethical responsibility is significantly associated with how individuals interpret moral situations and translate ethical knowledge into action‐oriented decisions [7]. According to SCT, individuals do not merely act based on knowledge but also based on internalised standards that guide self‐regulation and behavioural consistency. Moral intelligence provides the cognitive foundation for recognising ethical issues, evaluating moral alternatives and understanding appropriate courses of action in clinical practice.

Moral intelligence enhances nurses’ awareness of moral norms and strengthens their internal sense of duty towards patients and professional obligations [21]. This heightened sense of ethical responsibility serves as a key motivational mechanism through which moral intelligence is translated into behaviour, as it fosters a strong obligation to act in accordance with ethical standards [22]. In turn, ethical responsibility activates self‐regulatory processes that guide nurses to align their decisions with professional ethical expectations, even in complex or time‐pressured clinical environments [23]. Consequently, ethical responsibility functions as a mediating mechanism that explains the relationship between moral intelligence and ethical decision‐making. Despite extensive discussions on moral cognition and ethical behaviour in nursing literature [4, 22], the mediating role of ethical responsibility between moral intelligence and ethical decision‐making remains underexplored. However, empirical evidence suggests that moral intelligence is positively associated with ethical responsibility [24, 25]. In addition, ethical responsibility has been shown to significantly relate to ethical decision‐making processes [23, 26], indicating that individuals with stronger ethical responsibility are more likely to engage in sound and principled ethical judgements. Given that nurses frequently encounter morally complex and high‐stakes decisions, ethical responsibility is introduced as a critical mechanism linking moral intelligence to ethical decision‐making. Consequently, the author purported that:

H_2_: Ethical responsibility mediates the relationship between moral intelligence and ethical decision‐making.

### 2.4. Time Urgency as a Moderator in the Indirect Relationship

Time urgency refers to a situational pressure characterised by the perceived need to make decisions within constrained time limits, often under uncertainty, competing demands and reduced opportunity for reflection [8]. Such pressures are common in nursing environments, where rapid clinical judgements are frequently required to maintain patient safety and continuity of care. In the present study, time urgency is conceptualised as a contextual boundary condition that relates to the indirect relationship between moral intelligence and ethical decision‐making through ethical responsibility. According to SCT, individuals rely on internalised cognitive and moral mechanisms to regulate their behaviour. Moral intelligence provides the moral awareness and ethical principles necessary for evaluating situations, whereas ethical responsibility reflects an individual’s perceived obligation to act in accordance with those principles. However, SCT posits that the enactment of these internal mechanisms is shaped by environmental conditions [11]. Time urgency represents a situational constraint that may disrupt self‐regulatory processes by limiting opportunities for careful reflection and ethical deliberation. Consistent with this view, prior research suggests that time‐sensitive environments often impair ethical reasoning by encouraging reliance on heuristics and intuitive judgements rather than systematic analysis [22, 27]. Within healthcare settings, time pressure has been linked to rushed clinical judgements and a greater likelihood of decision errors, particularly when professionals are confronted with complex patient needs and competing responsibilities [28].

The moderating role of time urgency on the indirect relationship can be explained through several interconnected mechanisms. First, time pressure constrains the application of moral capacities in real‐time clinical situations, reducing nurses’ ability to engage in comprehensive ethical reasoning and evaluate the consequences of alternative courses of action [28]. As a result, the extent to which moral intelligence informs ethical decision‐making may be diminished. Second, heightened time urgency reduces the depth of ethical processing, increasing reliance on intuitive judgements and heuristic‐based decision‐making rather than deliberate ethical analysis [29]. Under these circumstances, nurses may prioritise immediate clinical demands over ethical considerations, thereby weakening the relationship between ethical responsibility and decision‐making [30]. Third, time urgency imposes cognitive strain that redirects attention toward speed and task completion rather than ethical accuracy, encouraging the use of decision shortcuts [31]. The depletion of cognitive resources associated with such conditions limits nurses’ ability to effectively apply their moral values and ethical responsibilities when making decisions. These mechanisms suggest that high levels of time urgency may weaken the indirect effect of moral intelligence on ethical decision‐making through ethical responsibility, as decisions become increasingly shaped by situational demands rather than deliberate ethical reflection.

Despite growing evidence regarding the effects of time pressure on judgement and decision‐making, limited research has examined its role in shaping the indirect pathway in which moral intelligence relates to ethical decision‐making via ethical responsibility. This gap is particularly evident within nursing contexts, where ethical responsibility constitutes a fundamental component of professional practice. Accordingly, the present study proposes the following hypothesis:

H_3_. Time urgency moderates the indirect association between moral intelligence and ethical decision‐making through ethical responsibility such that the indirect relationship is weaker when time urgency is high.

## 3. Methodology

### 3.1. Research Approach

A quantitative research approach was adopted to enable the objective examination of the associations among moral intelligence, ethical responsibility, time urgency and ethical decision‐making. This approach was considered appropriate as it facilitates statistical testing of hypothesised relationships and supports the generalisability of findings within similar clinical contexts [32]. The study was conducted among registered nurses working in selected public healthcare facilities in Ghana, a context characterised by increasing patient loads, complex clinical cases and heightened demands for rapid yet ethically sound decision‐making. These conditions make the setting suitable for examining how moral capacities translate into ethical behaviour under varying levels of situational pressure [1]. This enhances the external relevance of the findings, given that public hospitals represent primary points of care where ethical and time‐sensitive decisions are frequently required. The study adopted a two‐wave time‐lagged survey design to improve temporal ordering and reduce common method variance (CMV). This separation strengthens internal validity and supports more robust causal inferences in behavioural research contexts [33]. A multistage sampling technique involving cluster and convenience sampling was employed to facilitate systematic and context‐appropriate participant selection. In the first stage, public hospitals were grouped into clusters based on geographical location, and 15 hospitals were selected to capture variations in workload intensity, resource availability and clinical complexity [34]. In the second stage, convenience sampling was applied within the selected hospitals to recruit available nurses who were willing to participate and had at least one year of clinical experience. This approach ensured that participants had some exposure to ethical decision‐making situations in clinical practice, enhancing the relevance of their responses.

The attrition analysis showed no statistically significant differences between participants retained at Wave 2 and those lost to follow‐up across age, moral intelligence, ethical responsibility, ethical decision‐making and time urgency (all *p*s > 0.05), indicating no evidence of differential attrition based on the Wave 1 characteristics examined.

### 3.2. Sample Characteristics

The required sample size was determined using a Monte Carlo simulation, following established procedures for power analysis in mediation and moderated mediation models [35]. The simulation was conducted based on the expected relationships among moral intelligence, ethical responsibility, ethical decision‐making and time urgency. The results indicated that a minimum sample of 300 respondents would provide adequate statistical power for detecting the proposed direct, indirect and interaction effects. Accordingly, 300 respondents were targeted for data collection to ensure sufficient statistical power for testing the proposed moderated mediation model. Data collection was conducted in two phases to ensure temporal separation of constructs. The first wave occurred in January 2026, during which structured questionnaires were administered face‐to‐face to collect demographic data, moral intelligence and time urgency. This approach enhanced response accuracy and reduced missing data. All 330 questionnaires were retrieved and deemed usable. The second wave was conducted in April 2026, during which participants completed an online questionnaire measuring ethical responsibility and ethical decision‐making. A total of 268 responses were received; however, after rigorous data cleaning procedures, including the removal of incomplete and inconsistent responses, 261 (87%) questionnaires were retained for final analysis. Time urgency was measured during Phase 1 because it reflects a relatively stable workplace condition rather than an immediate outcome. Assessing it earlier also helped temporally separate the moderator from the mediator and outcome variables, thereby reducing potential common method bias (CMB) [36]. The study included nurses from medical, surgical, emergency, outpatient, maternity, paediatric and intensive care units across the participating hospitals. Most respondents were female (81.7%), while 62.5% had attained at least a tertiary‐level qualification. The respondents had a mean age of 37.6 years, and the majority had at least 10 years of clinical experience. Nurses working in the maternity unit constituted the largest proportion of the sample (41.8%). Across the participating units, nurse‐to‐patient ratios ranged from 1:5 to 1:12, reflecting differences in patient workload and staffing requirements. These characteristics are presented in Tables 1 and 2. Among the demographic variables, age and job position were included as control variables due to evidence in prior research suggesting their potential influence on both moral intelligence and ethical decision‐making [16, 37].

**TABLE 1:** Attrition analysis comparing participants retained and lost to follow‐up.

Wave 1 characteristic	Retained (*n* = 268)	Lost to follow‐up (*n* = 62)	*t*	*p*
Age	37.58 ± 5.72	37.06 ± 5.72	0.65	0.520
Moral intelligence	3.01 ± 0.82	3.24 ± 0.91	−1.83	0.071
Ethical responsibility	2.97 ± 0.61	2.82 ± 0.67	1.61	0.110
Ethical decision‐making	3.62 ± 1.18	3.51 ± 1.06	0.72	0.473
Time urgency	3.45 ± 1.23	3.66 ± 1.21	−1.23	0.223

*Note:* Values are presented as M ± SD. *t* = independent‐samples *t* statistic.

**TABLE 2:** Demographic characteristics of respondents (*N* = 261).

Demographic characteristics	Category	Frequency (*n*)	Percentage (%)
Gender	Female	213	81.6
	Male	48	18.4

Educational qualification	Tertiary level or above	163	62.5
	Below tertiary level	98	37.5

Age	Mean age	261	37.6 years

Professional experience	Less than 10 years	77	29.5
	10 years or more	184	70.5

Department/unit	Other units	152	58.2
	Maternity	109	41.8

Total		261	100.0

Data analysis was performed using Hayes’ PROCESS Macro, which is suitable for testing moderated mediation models as it allows simultaneous estimation of direct, indirect and interaction effects within a single framework.

### 3.3. Measures

Responses for all variables were measured using a five‐point Likert scale ranging from 1 (*strongly disagree*) to 5 (*strongly agree*). Moral intelligence was measured using the 7‐item Moral Intelligence Scale developed by Khampa [38]. The scale assesses the extent to which nurses demonstrate moral awareness, integrity, responsibility and ethical behaviour in professional practice. A sample item includes: “I act according to what I believe is morally right, even when it is difficult.” The scale demonstrated strong internal consistency, with a Cronbach’s alpha of 0.877 in this study. Ethical decision‐making was assessed using the 10‐item scale developed by Dufrene and Glosoff [39]. The scale measures the cognitive process of evaluating ethical dilemmas and selecting appropriate courses of action based on professional ethical standards. A sample item includes: “I consider all possible consequences before making ethical decisions in my work.” The scale demonstrated acceptable reliability (*α* = 0.902).

Ethical responsibility was measured using the 6‐item scale developed by Anita et al. [40]. The scale captures individuals perceived moral obligation to act ethically, ensure accountability and uphold professional ethical standards. A representative item is “I feel personally accountable for the ethical outcomes of my nursing decisions.” The scale showed an acceptable internal consistency (*α* = 0.890) in this study. Time urgency was also gauged using the 10‐item Time Urgency Scale developed by Landy et al. [41]. The scale assesses the degree to which individuals experience pressure to act quickly, meet tight deadlines and make rapid decisions. An item on the scale is “I feel pressured to complete tasks as quickly as possible.” The scale demonstrated acceptable reliability (*α* = 0.885) in this study (see Table 3).

**TABLE 3:** Factor loadings, internal consistency and average variance extracted (AVE) estimates.

Items	Loadings	% Variance exp.	CR	VIF
Moral intelligence (MI; *α* = 0.877)		23.726	0.893	2.150
MI‐2	0.911^∗∗^			
MI‐7	0.903^∗∗^			
MI‐4	0.885^∗∗^			
MI‐1	0.882^∗∗^			
MI‐3	0.860^∗∗^			
MI‐6	0.849^∗∗^			
Ethical decision‐making (EDM; *α* = 0.902)		17.883	0.900	1.733
EDM‐6	0.926^∗∗^			
EDM‐4	0.923^∗∗^			
EDM‐1	0.916^∗∗^			
EDM‐9	0.914^∗∗^			
EDM‐2	0.903^∗∗^			
EDM‐3	0.890^∗∗^			
EDM‐10	0.884^∗∗^			
EDM‐5	0.886^∗∗^			
EDM‐8	0.882^∗∗^			
EDM‐7	0.858^∗∗^			
Ethical responsibility (ER; *α* = 0.890)		11.925	0.897	2.194
ER‐4	0.912^∗∗^			
ER‐1	0.905^∗∗^			
ER‐6	0.893^∗∗^			
ER‐2	0.861^∗∗^			
ER‐5	0.834^∗∗^			
ER‐3	0.788^∗∗^			
Time urgency (TU; *α* = 0.885)		10.028	0.897	3.021
TU‐4	0.911^∗∗^			
TU‐1	0.903^∗∗^			
TU‐9	0.894^∗∗^			
TU‐7	0.880^∗∗^			
TU‐2	0.877^∗∗^			
TU‐10	0.859^∗∗^			
TU‐3	0.838^∗∗^			
TU‐6	0.818^∗∗^			
TU‐5	0.788^∗∗^			

*Note:* KMO = 0.809. Source: Field survey (2026).

^∗∗^Satisfactory indicator loadings.

### 3.4. Ethical Consideration

Ethical approval was obtained from the Ghana Communication Technology University Review Board (Approval No. GCTU/EREA/0174; approved on 9 November 2025) by the corresponding author, Prince Addai, before data collection commenced. Participation was voluntary, and informed consent was obtained from all respondents. Confidentiality and anonymity were strictly maintained throughout the research process, and all procedures were conducted in accordance with established ethical standards for research involving human participants.

### 3.5. Evaluation of Factor Structure and Sample Suitability

Prior to hypothesis testing, the measurement items were subjected to principal component analysis (PCA) with Varimax rotation to examine the underlying component structure and assess the adequacy of the measurement items. Components with eigenvalues greater than 1 were retained. The retained items loaded appropriately on their respective constructs, with most component loadings exceeding the recommended benchmark of 0.70 [42]. Of the 33 items initially selected, 31 met the required loading criteria and were retained for the final analysis, while two items were removed due to inadequate loadings (see Tables 3 and 4). The retained indicators demonstrated satisfactory representation of their corresponding constructs. The suitability of the data for PCA was assessed using the Kaiser–Meyer–Olkin (KMO) measure and Bartlett’s test of sphericity. The KMO coefficient of 0.809 indicated adequate sampling adequacy for component analysis. Bartlett’s test of sphericity was statistically significant (*χ* ^2^ = 1152.016, *p* < 0.001), confirming that the correlation matrix was suitable for component analysis. The retained components jointly explained 63.562% of the total variance, indicating that the component solution accounted for a substantial proportion of the variance in the measurement items.

**TABLE 4:** Fit indices of the constructs.

Model		*χ* ^2^/df	CFI	TLI	RMSEA
(1)	Four‐factor model	1.905	0.912	0.904	0.032
(a)	Moral intelligence	2.803	0.890	0.897	0.048
(b)	Ethical responsibility	2.116	0.903	0.902	0.035
(c)	Ethical decision making	1.971	0.910	0.900	0.031
(d)	Time urgency	2.928	0.884	0.873	0.050
(2)	One‐factor model	8.490	0.739	0.688	0.149

*Note:* *χ* ^2^/df = chi‐square/degrees of freedom.

Abbreviations: CFI, comparative fit index; RMSEA, root mean square error of approximation; TLI, Tucker–Lewis index.

Following PCA, confirmatory factor analysis (CFA) was conducted to validate the measurement model. CFA was performed using JASP 0.18.3 with the maximum likelihood estimation method. The analysis examined the factor structure of the study constructs using model fit indices, including chi‐square (*χ* ^2^), degrees of freedom (df), chi‐square to degrees of freedom ratio (*χ* ^2^/df), comparative fit index (CFI), Tucker–Lewis index (TLI), root mean square error of approximation (RMSEA) and standardised root mean square residual (SRMR). Convergent validity was assessed using factor loadings, composite reliability (CR) and average variance extracted (AVE), while discriminant validity was evaluated using the Fornell–Larcker criterion and comparison of AVE values with shared correlations. PCA and CFA were conducted using the same sample. This is acknowledged as a limitation because using the same dataset for both exploratory and confirmatory analyses provide weaker validation than testing the measurement structure using independent samples.

### 3.6. Internal Consistency, Validity Assessment and Data Assumptions

The reliability of the constructs was evaluated using CR. All CR values exceeded the recommended threshold of 0.70, indicating satisfactory internal consistency among the measurement items [43]. Convergent validity was further established, as the AVE for all constructs surpassed the minimum criterion of 0.50, demonstrating that the indicators adequately captured the variance of their respective latent constructs. To establish discriminant validity, the maximum shared variance (MSV) values were compared with the AVE values for each construct [42]. The results indicated that the AVE values exceeded the corresponding MSV values across all constructs, confirming that the variables captured distinct theoretical concepts (see Table 5). The distributional properties of the data were also examined to ensure compliance with the assumptions of parametric analysis. Both the Kolmogorov–Smirnov and Shapiro–Wilk tests were not significant, indicating that the data approximated normal distribution patterns. Furthermore, the correlation matrix showed that the intervariable correlations were below 0.70. This suggests the absence of multicollinearity among the constructs [44].

**TABLE 5:** Descriptive and correlation analysis (*n* = 261).

	Constructs	Mean	SD	Correlations			MSV	AVE
				1	2	3		
1	Moral intelligence	3.11	0.97	—			0.203	0.713
3	Ethical responsibility	2.98	0.70	0.38^∗∗^	—		0.160	0.736
3	Ethical decision‐making	3.02	1.15	0.45^∗∗^	0.31^∗∗^	—	0.203	0.640
4	Time urgency	3.56	1.22	−0.33^∗∗^	−0.40^∗∗^	−0.32^∗∗^	0.160	0.608

^∗∗^ *p* < 0.01.

Furthermore, potential multicollinearity issues among the study variables were evaluated using the variance inflation factor (VIF). The obtained VIF values remained below the recommended threshold of 5, indicating that the predictor variables were sufficiently distinct and that multicollinearity was unlikely to distort the regression estimates (Hair et al., 2017).

### 3.7. Assessing CMV and Interconstruct Relationships

Given that the data were collected using self‐report measures, procedural and statistical remedies were undertaken to assess the potential influence of CMB. Procedurally, psychological separation was introduced through the time‐lagged research design, whereby the predictor and criterion variables were measured at separate time points, thereby reducing the likelihood that respondents would provide consistent responses across measures. Statistically, the hypothesised four‐factor measurement model was compared with a competing single‐factor model. The hypothesized four‐factor measurement model demonstrated an acceptable fit to the data (*χ* ^2^/df = 1.905, CFI = 0.912, TLI = 0.904, RMSEA = 0.032), whereas the one‐factor model showed substantially poorer fit (*χ* ^2^/df = 8.490, CFI = 0.739, TLI = 0.688, RMSEA = 0.149). The substantially poorer fit of the one‐factor model supports the distinctiveness of the study constructs and suggests that CMB was unlikely to substantially influence the observed relationships. In addition, Harman’s single‐factor assessment indicated that no single factor accounted for more than 50% of the total variance, providing further evidence against a dominant common method factor.

## 4. Results

### 4.1. Correlation Analysis

Correlation analysis further revealed significant associations among the variables (see Table 5). Table 5 presents the descriptive statistics and correlations among the study variables. Moral intelligence was positively correlated with ethical responsibility (*r* = 0.38, *p* < 0.01) and ethical decision‐making (*r* = 0.45, *p* < 0.01). Ethical responsibility was also positively correlated with ethical decision‐making (*r* = 0.31, *p* < 0.01). In contrast, time urgency was negatively correlated with moral intelligence (*r* = −0.33, *p* < 0.01), ethical responsibility (*r* = −0.40, *p* < 0.01) and ethical decision‐making (*r* = −0.32, *p* < 0.01). These correlations indicate that higher moral intelligence and ethical responsibility were associated with higher ethical decision‐making, whereas greater time urgency was associated with lower scores on all three constructs. The correlation results provide preliminary evidence of associations among the variables consistent with the proposed model.

### 4.2. Examination of Direct, Mediating and Conditional Effects

To examine the proposed hypotheses, PROCESS Model 4 was used to assess the direct and mediating associations, while Model 7 was applied to test the moderated mediation model. The direct association between moral intelligence and ethical decision‐making was positive and statistically significant (*B* = 0.327, SE = 0.041, 95% CI [0.247, 0.407]), providing support for H1. Ethical responsibility also mediated the association between moral intelligence and ethical decision‐making, as the indirect estimate was statistically significant (*B* = 0.109, BootSE = 0.027, 95% CI [0.056, 0.162]). The exclusion of zero from the bootstrap confidence interval provided support for H2 (see Table 6).

**TABLE 6:** Direct, indirect and moderated mediation associations.

Hypothesis	Association	B	SE	BootLLCI	BootULCI
H1	MI ⟶ EDM	0.327	0.041	0.247	0.407
H2	MI ⟶ ER ⟶ EDM	0.109	0.027	0.056	0.162
H3	Index of moderated mediation	−0.115	0.022	−0.158	−0.072

*Note:* Indirect and moderated mediation estimates are based on 5000 bootstrap samples. Statistical significance is indicated when the 95% bootstrap confidence interval does not include zero.

Abbreviations: BootLLCI, bootstrap lower limit confidence interval; BootULCI, bootstrap upper limit confidence interval; EDM, ethical decision‐making; ER, ethical responsibility; MI, moral intelligence.

The conditional indirect associations are presented in Table 7. The index of moderated mediation was negative and statistically significant (Index = −0.115, BootSE = 0.022, 95% CI [−0.158, −0.072]), indicating that the indirect association between moral intelligence and ethical decision‐making through ethical responsibility differed according to the level of time urgency. Specifically, the indirect association was strongest under low time urgency (*B* = 0.214, 95% CI [0.116, 0.312]), followed by the mean level (*B* = 0.181, 95% CI [0.109, 0.253]), and was weakest under high time urgency (*B* = 0.109, 95% CI [0.029, 0.189]). These findings suggest that greater time urgency corresponds with a weaker indirect association through ethical responsibility, supporting H3.

**TABLE 7:** Conditional indirect associations at levels of time urgency.

Time urgency	Effect	BootSE	BootLLCI	BootULCI
Low (−1 SD)	0.214	0.050	0.116	0.312
Mean	0.181	0.037	0.109	0.253
High (+1 SD)	0.109	0.041	0.029	0.189

*Note:* Conditional indirect associations are based on 5000 bootstrap samples.

Abbreviations: BootLLCI, bootstrap lower limit confidence interval; BootSE, bootstrap standard error; BootULCI, bootstrap upper limit confidence interval.

## 5. Discussion

The present study examined the moderated mediation roles of ethical responsibility and time urgency in the associations between moral intelligence and ethical decision‐making among nurses. First, the study revealed that moral intelligence was positively and significantly related to ethical decision‐making among nurses. This suggests that nurses who possess higher levels of moral intelligence are more likely to recognise ethical obligations, evaluate moral consequences and make decisions that align with professional ethical standards. In the nursing context, moral intelligence serves as an internal moral capacity that enables nurses to distinguish appropriate from inappropriate actions and apply ethical principles when confronted with complex clinical situations [19]. This finding supports the assumptions of SCT, which proposes that individuals’ behaviour is influenced by internal cognitive processes, moral standards and self‐regulatory mechanisms [10]. Specifically, nurses with stronger moral intelligence are better positioned to regulate their actions according to ethical values, even when faced with competing demands and challenging healthcare circumstances [18]. This finding is consistent with previous studies indicating that individuals with stronger moral capacities demonstrate greater ethical awareness and engage more frequently in ethical behaviours within organisational contexts [5, 19]. One possible explanation is that morally intelligent nurses are more likely to carefully consider the ethical implications of their decisions, prioritise patient welfare and act in accordance with professional responsibilities. Consequently, moral intelligence becomes an important psychological resource that strengthens ethical decision‐making in nursing practice [18].

The second finding demonstrated that ethical responsibility significantly mediates the relationship between moral intelligence and ethical decision‐making. This indicates that moral intelligence contributes to ethical decision‐making partly by strengthening nurses’ sense of ethical duty and accountability. The finding highlights ethical responsibility as an important psychological mechanism through which moral intelligence is linked to ethical decision‐making. The result is consistent with SCT, which posits that internalised moral standards influence behavioural outcomes through self‐regulatory mechanisms [10]. In this framework, ethical responsibility functions as the mediating mechanism through which moral intelligence is translated into ethical decision‐making. Thus, ethical responsibility facilitates the conversion of moral values into observable ethical behaviour by motivating nurses to act in accordance with professional obligations and established moral expectations [23]. This finding may be explained by the tendency of nurses who feel ethically responsible to engage in careful moral reflection before making decisions, particularly in complex clinical situations. Such individuals are also likely to experience stronger moral accountability, making them less likely to compromise ethical standards in professional practice [23, 26]. Importantly, the observed effect should be interpreted not only in terms of statistical significance but also in terms of practical relevance. Although the findings indicate a meaningful relationship, the extent to which these effects translate into clinically meaningful improvements in ethical decision‐making should be interpreted cautiously, as the study did not directly assess patient‐level outcomes or clinical changes resulting from improved ethical decision‐making.

The final finding showed that time urgency weakens the indirect association between moral intelligence and ethical decision making through ethical responsibility. This finding supports SCT, which suggests that environmental conditions can influence how internal cognitive mechanisms are translated into behaviour [10]. In this study, time urgency functions as an environmental constraint that conditions the relationship between moral intelligence and ethical responsibility, thereby weakening the subsequent indirect association with ethical decision making. Under high time urgency, nurses may experience reduced cognitive flexibility and fewer opportunities for reflective ethical reasoning [28], which may constrain their ability to convert moral intelligence into ethical responsibility. A possible explanation is that heightened time pressure shifts attention toward immediate task completion and rapid responses, leaving less opportunity for deliberate moral evaluation and the development of ethical responsibility before making clinical decisions (Wu et al., 2022).

### 5.1. Limitations and Research Suggestions

This study has numerous limitations that should be considered when interpreting the results. First, the study relied on self‐reported survey questionnaires, which may increase the likelihood of CMV due to respondents’ tendencies toward social desirability and consistent responses. In particular, self‐reported measures of ethical decision‐making may have been influenced by social desirability bias, as nurses may have been inclined to portray themselves as behaving more ethically than they do in actual clinical practice. Although the time‐lagged design reduces temporal overlap and common method concerns, the observational nature of the study does not allow definitive causal conclusions. Second, the use of a convenience sampling technique limits the generalisability of the findings because the sample may not fully represent all nurses within Ghana’s healthcare sector. Future studies may, therefore, employ probability sampling approaches to improve representativeness and external validity. Third, the study focused exclusively on nurses working in public healthcare facilities, restricting the applicability of the findings to other healthcare professionals and organisational settings. Future research may replicate the study among doctors, pharmacists and other healthcare practitioners, as well as within private healthcare institutions. Finally, although ethical responsibility and time urgency were examined as the mediating and moderating mechanisms, respectively, other psychological and contextual variables may also explain the relationship between moral intelligence and ethical decision‐making. Future studies may, therefore, consider additional mediators and moderators such as moral courage, ethical climate, emotional exhaustion, and workload pressure to provide a more comprehensive understanding of ethical decision‐making processes in healthcare environments.

### 5.2. Implications and Conclusions

The findings of this study make important contributions to the existing literature on moral intelligence and ethical decision‐making within healthcare settings. Although previous studies have examined ethical behaviour among healthcare professionals, limited attention has been given to the psychological mechanisms through which moral intelligence relates to ethical decision‐making among nurses, particularly in the African context. By introducing ethical responsibility as a mediating mechanism, this study extends current literature by demonstrating that moral intelligence does not only relate to ethical decision‐making alone but also operates through nurses’ sense of ethical obligation and accountability. Additionally, the inclusion of time urgency as a moderating condition advances the understanding of how situational pressures shape ethical processes in clinical environments. The study, therefore, fills a significant gap by integrating personal moral capacities, psychological responsibility and contextual pressures within a single explanatory framework of ethical decision‐making among nurses.

The findings also have important implications for SCT. SCT explains behaviour as the product of reciprocal interactions among personal cognitive factors, self‐regulatory processes and environmental influences [10]. The observed positive association between moral intelligence and ethical responsibility is consistent with this perspective, as it reflects the role of internal moral standards in shaping self‐regulation. In addition, ethical responsibility serves as a key mechanism through which moral intelligence relates to ethical decision‐making, showing how self‐regulatory processes guide behaviour in line with internalised standards. The findings further indicate that environmental conditions influence the strength of these internal processes. In particular, time urgency shapes the extent to which moral cognition and ethical responsibility are effectively expressed in decision‐making contexts. High levels of time pressure appear to constrain cognitive resources and reduce opportunities for reflective ethical reasoning, thereby limiting the full expression of internal moral capacities in practice.

The findings provide several potential practical implications for healthcare institutions, nursing administrators and policymakers. First, the positive association between moral intelligence and ethical decision‐making suggests that ethics‐based professional development may be relevant for strengthening nurses’ integrity, empathy, honesty and moral reasoning. Nursing education and professional training programmes could, therefore, consider incorporating moral intelligence development modules and reflective learning activities to support nurses’ ethical awareness and judgement. Second, the mediating role of ethical responsibility indicates that ethical responsibility represents an important psychological mechanism through which nurses’ moral capacities are associated with ethical decision‐making. Healthcare institutions may, therefore, consider approaches such as ethical mentoring, ethical leadership and clearly communicated professional standards to reinforce ethical accountability and responsibility. Creating an organisational culture that emphasises ethical responsibility may support nurses in internalising ethical obligations and applying them consistently during patient care. Third, the moderated mediation findings indicate that higher time urgency is associated with a weaker indirect relationship between moral intelligence and ethical decision‐making through ethical responsibility. This finding suggests that excessive workload pressures and staffing constraints may warrant attention in clinical environments. Potential strategies could include reviewing nurse‐to‐patient ratios, shift schedules, decision support systems and staffing arrangements to determine whether they can provide nurses with sufficient time for reflection and ethical evaluation. In departments where high time pressure is unavoidable, setting specific support mechanisms may also be considered. However, these strategies were not evaluated in the present study and, therefore, require prospective evaluation.

The findings also have potential implications for nursing leadership, ethics education and policy implementation. For nursing leadership, nurse managers and clinical leaders may foster ethical work environments by modelling ethical conduct, encouraging open discussions of ethical dilemmas and providing guidance on ethical decision‐making, particularly in high‐pressure clinical situations. Regarding ethics education, nursing schools and continuing professional development programmes could complement theoretical ethics instruction with case‐based learning, simulation exercises and reflective practice activities that develop moral intelligence and ethical responsibility in clinical contexts. From a policy perspective, healthcare institutions and regulatory bodies may consider whether ethical responsibility and moral intelligence could be incorporated into professional competency frameworks, performance appraisal systems and organisational ethics policies. Similarly, policies addressing staffing levels, time pressure and access to ethical consultation may be considered as potential approaches for supporting ethical decision‐making. The effectiveness of these approaches, however, should be established through future empirical evaluation.

In conclusion, this study demonstrates an association between moral intelligence and ethical decision‐making among nurses, particularly through ethical responsibility, while time urgency conditions this indirect relationship. The findings suggest that ethical decision‐making may reflect both individual moral capacities and situational conditions within the clinical environment. Accordingly, strengthening ethical practice may require attention to both nurses’ moral capacities and workplace conditions that support reflective and ethically responsible decision‐making under professional pressure. Further research is needed to determine whether specific organisational and policy interventions can improve these outcomes.

## Author Contributions

Ethel Yiranbon Annor‐Tenkorang: conceptualisation of the study, development of research objectives, data analysis and approved submission for publication.

Prince Addai: literature review, design of methodology, data analysis, interpretation of results and discussion.

Rejoice Mottey: assisted with data collection and wrote the draft work.

James Abadektemi Abasimi: provided statistical guidance, reviewed the final manuscript and approved submission for publication.

Emmanuel Atta Kumah Amponsah: supervision of the fieldwork, checking through the references and reviewing the final manuscript.

## Funding

This research received no external funding. The study was fully self‐financed by the authors, and no grants or institutional funding were obtained for its execution.

## Conflicts of Interest

The authors declare no conflicts of interest.

## Data Availability

The data that support the findings of this study are available from the corresponding author upon reasonable request. The data are not publicly available due to ethical considerations involving participant confidentiality.
